# Cortical Dynamics of Figure-Ground Separation in Response to 2D Pictures and 3D Scenes: How V2 Combines Border Ownership, Stereoscopic Cues, and Gestalt Grouping Rules

**DOI:** 10.3389/fpsyg.2015.02054

**Published:** 2016-01-26

**Authors:** Stephen Grossberg

**Affiliations:** ^1^Center for Adaptive Systems, Graduate Program in Cognitive and Neural Systems, Center for Computational Neuroscience and Neural Technology, Boston UniversityBoston, MA, USA; ^2^Department of Mathematics, Boston UniversityBoston, MA, USA

**Keywords:** figure-ground separation, border ownership, cortical area V2, boundary grouping, surface filling-in, Gestalt rules, FACADE model, 3D LAMINART model

## Abstract

The FACADE model, and its laminar cortical realization and extension in the 3D LAMINART model, have explained, simulated, and predicted many perceptual and neurobiological data about how the visual cortex carries out 3D vision and figure-ground perception, and how these cortical mechanisms enable 2D pictures to generate 3D percepts of occluding and occluded objects. In particular, these models have proposed how border ownership occurs, but have not yet explicitly explained the correlation between multiple properties of border ownership neurons in cortical area V2 that were reported in a remarkable series of neurophysiological experiments by von der Heydt and his colleagues; namely, border ownership, contrast preference, binocular stereoscopic information, selectivity for side-of-figure, Gestalt rules, and strength of attentional modulation, as well as the time course during which such properties arise. This article shows how, by combining 3D LAMINART properties that were discovered in two parallel streams of research, a unified explanation of these properties emerges. This explanation proposes, moreover, how these properties contribute to the generation of consciously seen 3D surfaces. The first research stream models how processes like 3D boundary grouping and surface filling-in interact in multiple stages within and between the V1 interblob—V2 interstripe—V4 cortical stream and the V1 blob—V2 thin stripe—V4 cortical stream, respectively. Of particular importance for understanding figure-ground separation is how these cortical interactions convert computationally complementary boundary and surface mechanisms into a consistent conscious percept, including the critical use of surface contour feedback signals from surface representations in V2 thin stripes to boundary representations in V2 interstripes. Remarkably, key figure-ground properties emerge from these feedback interactions. The second research stream shows how cells that compute absolute disparity in cortical area V1 are transformed into cells that compute relative disparity in cortical area V2. Relative disparity is a more invariant measure of an object's depth and 3D shape, and is sensitive to figure-ground properties.

## Introduction

### 1. Explaining figure-ground separation as a consequence of complementary consistency

The FACADE (Form-And-Color-And-DEpth) model (e.g., Grossberg, 1987, 1994, 1997, 2014, in press; Grossberg and McLoughlin, 1997; Pinna and Grossberg, 2005; Grossberg and Hong, 2006; Grossberg et al., 2007), and its further development and extension by the 3D LAMINART model (e.g., Grossberg, 1999; Grossberg and Swaminathan, 2004; Grossberg and Yazdanbakhsh, 2005; Cao and Grossberg, 2005, 2012; Grossberg et al., 2008; Fang and Grossberg, 2009; Léveillé et al., 2010), have explained and predicted many psychological and neurobiological data about 3D vision and figure-ground perception. These models embody a fundamental property of global brain organization; namely, that advanced brains are organized into parallel cortical processing streams with *complementary* properties (Grossberg, 2000): to process certain combinations of properties, each cortical stream cannot process computationally complementary properties. Interactions between these streams, across multiple processing stages, overcome their complementary deficiencies to compute effective representations of the world. For the case of vision, these interactions convert boundary and surface computations that obey complementary laws into a consistent percept, thereby achieving the property of *complementary consistency*. These properties are reviewed here to provide a self-contained exposition. They are also reviewed in Grossberg (2014) with a different explanatory goal in mind.

#### Complementary properties of boundary completion and surface filling-in

**Figure 1:**
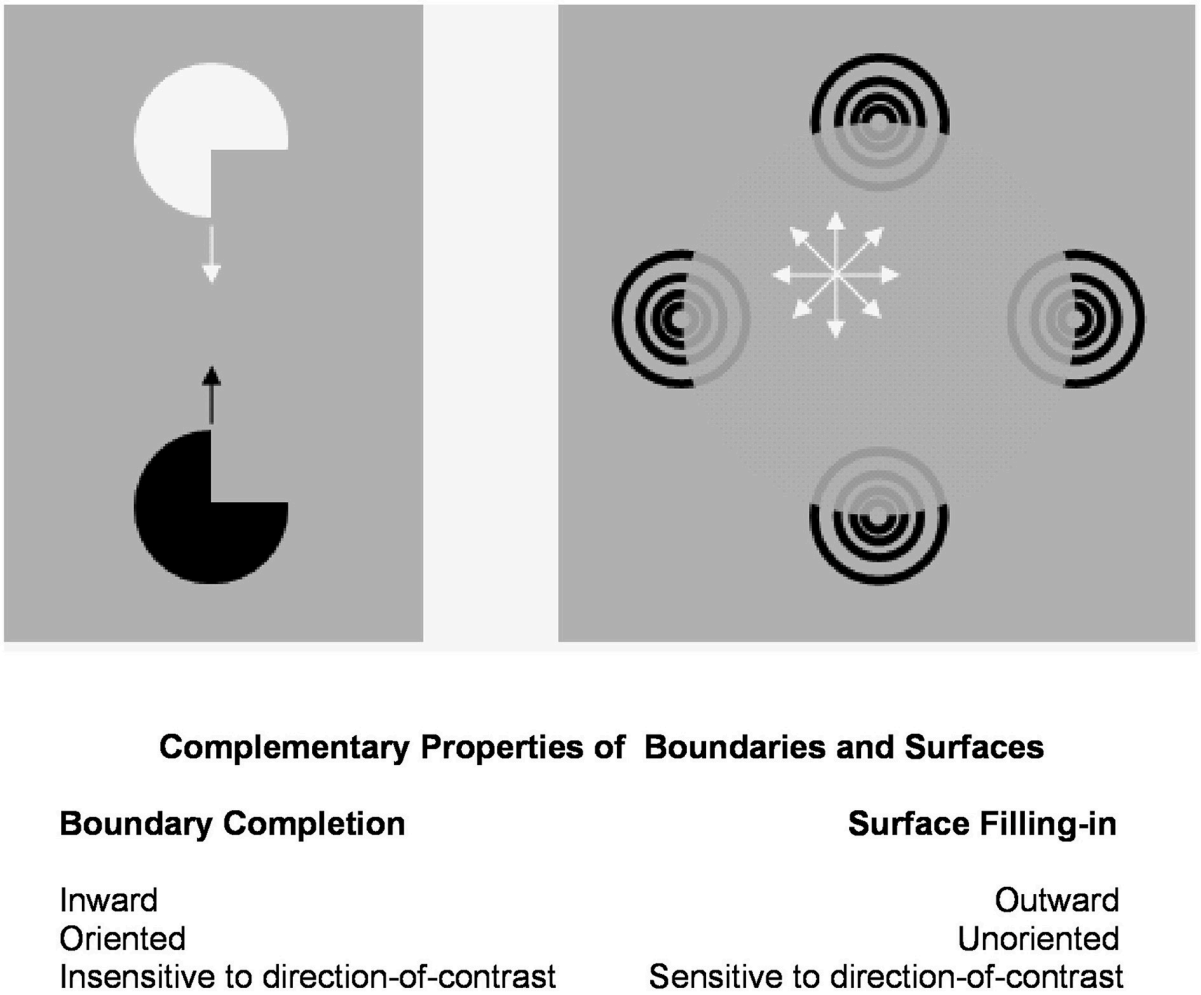
**Boundary completion and surface filling-in obey computationally complementary laws**. Boundaries complete *inwardly* in an *oriented* manner in response to pairs or greater numbers of inducers. Boundary completion also pools across opposite contrast polarities, and thus forms in a manner that is *insensitive* to contrast polarity. As a result, “all boundaries are invisible.” In contrast, surface filling-in spreads *outwardly* from each feature contour inducer in an *unoriented* manner and does not pool opposite contrast polarities, hence is *sensitive* to contrast polarity. As a result, all conscious percepts of visual qualia are surface percepts, including percepts of such seemingly simple stimuli as dots or lines, which also generate boundary groupings that contain filling-in of their surface brightnesses and/or colors; cf., simulations in Grossberg and Mingolla (1985b).

Figure 1 summarizes complementary properties whereby boundary groupings are completed and surfaces are filled-in with brightness and/or color.

**Figure 2:**
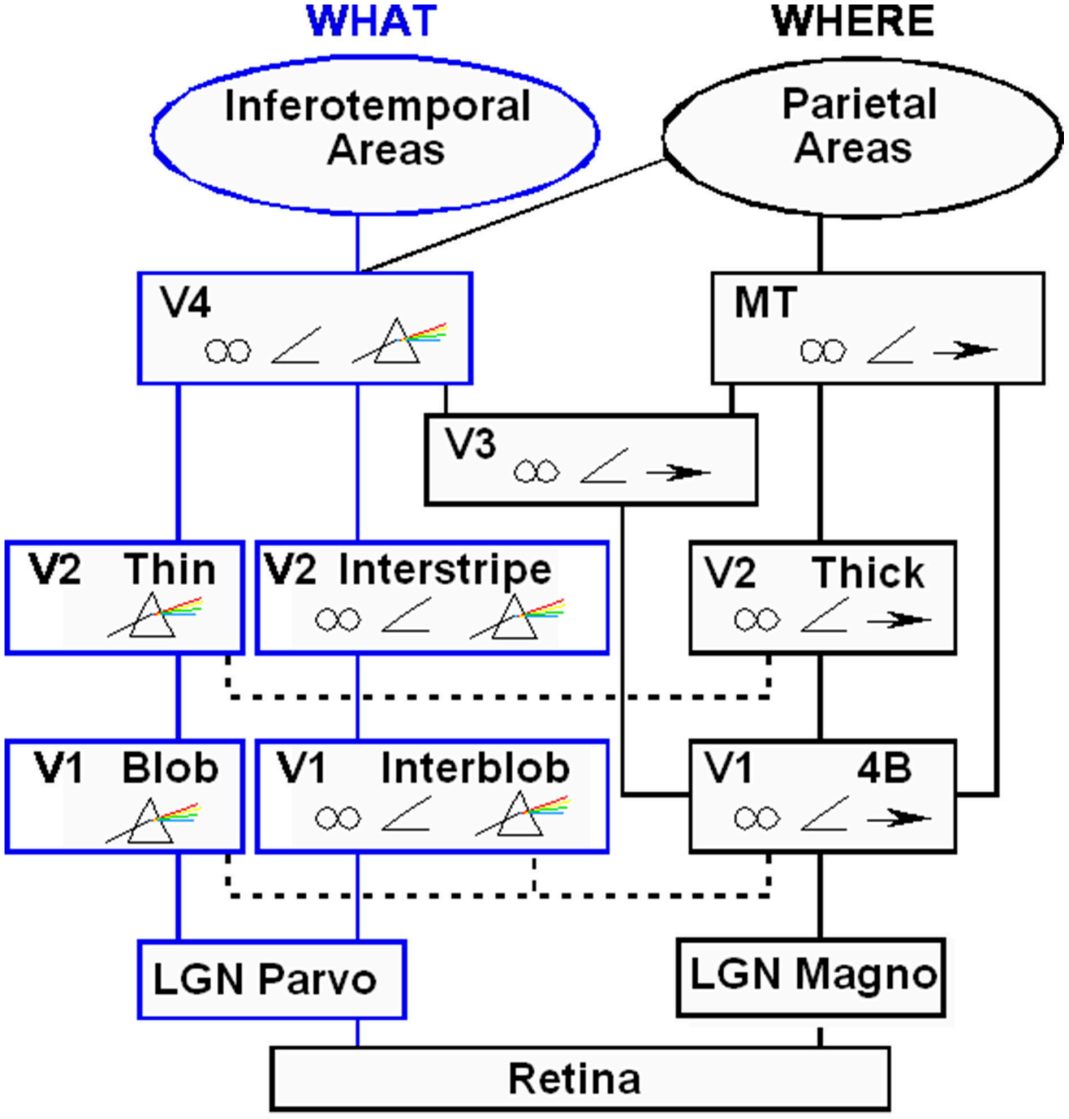
**Anatomical connections and receptive field properties of early visual areas in the macaque monkey**. LGN, Lateral Geniculate Nucleus; V1, striate visual cortex; V2, V3, V4, MT, prestriate cortical areas. The boundary stream goes through the blobs and thin stripes to cortical area V4 and inferotemporal areas. The surface stream goes through interblobs and interstripes to V4. The motion stream goes through V1 and MT to the parietal areas. Prism, wavelength selectivity; angle symbol, orientation selectivity; spectacles, binocular selectivity; and right-pointing arrow, selectivity to motion in a prescribed direction. Reprinted with permission from DeYoe and Van Essen (1988).

Boundaries are completed in the cortical stream from V1 interblobs to V2 interstripes and on to V4, whereas surfaces are filled-in in the cortical stream from V1 blobs to V2 thin stripes and on to V4 (Figure 2). These properties are more thoroughly described, along with perceptual and neurobiological data that support them, in a series of earlier articles; e.g., Grossberg (1994, 1997, 2003). They are briefly reviewed here for completeness.

**Figure 3:**
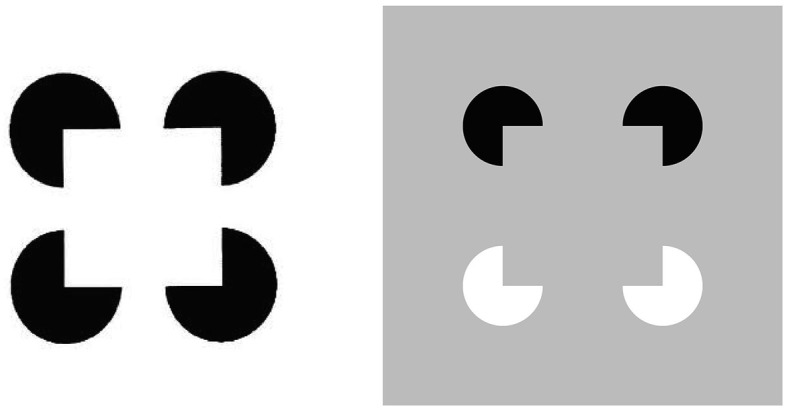
**Kanizsa square (left panel) and reverse-contrast Kanizsa square (right panel)**. The Kanizsa square appears brighter than its background due to the brightness induction by the four black pac man figures. In contrast, the reverse-contrast Kanizsa square may be recognized, but not seen, if the brightness induction by the black-to-gray pac man inducers balances the darkness induction due to the white-to-gray pac man inducers after filling-in.

All perceptual boundaries are completed *inwardly* between pairs or greater numbers of inducers. This completion process proceeds in an *oriented* fashion, as illustrated by how pairs of collinear pacman edges in Figure 3 induce completion of a colinear illusory contour between them. Boundaries are also *insensitive* to contrast polarity, because they pool input signals over opposite contrast polarities at each position. This pooling property is illustrated by a reverse-contrast Kanizsa square (Figure 3). During perception of natural scenes, polarity-pooling enables a boundary to form continuously along the bounding contour of a surface that lies in front of a background whose relative contrasts reverse along the boundary's perimeter. The pooling property led to the prediction that “all boundaries are invisible” (Grossberg, 1984, 1994) since, by pooling over opposite contrast polarities at each position, boundaries cannot represent a visible contrast difference.

**Figure 4:**
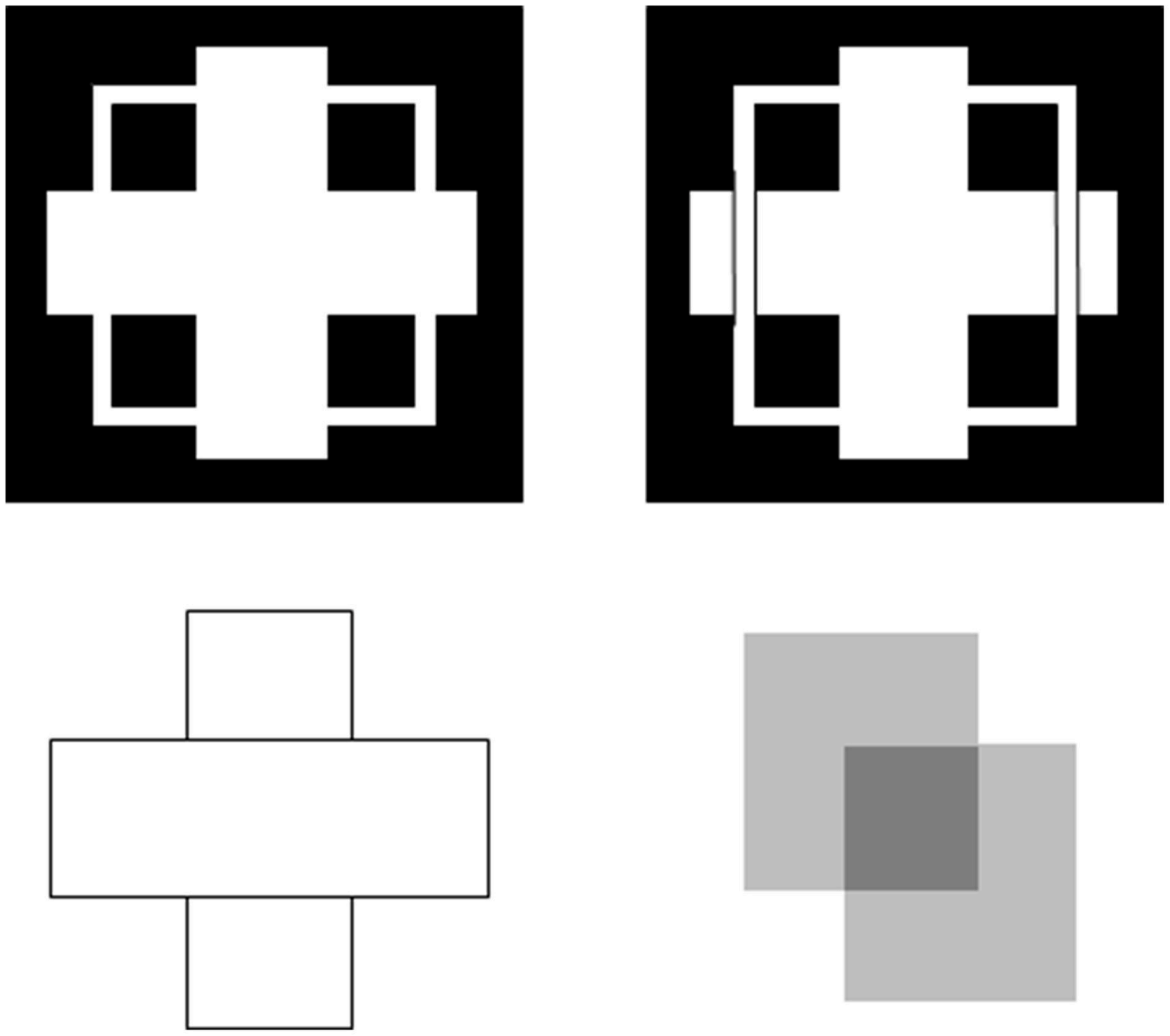
**T-junctions influence figure-ground percepts**. The two figures in the top row illustrate Kanizsa stratification. In the left panel, the white cross appears in front of the square border most of the time. The white in positions where the cross occludes the square appears to belong to the cross, and is in front of the square, which is amodally completed behind it. On occasion, the percept flips with the square appearing in front of the cross. Then the white area that previously belonged to the cross appears to belong to the square, with the cross amodally completed behind it. In the right panel, even when the extra black vertical lines force the vertical square bar to always appear in front of the cross, the horizontal branches of the square are amodally recognized behind the vertical bars of the cross, leading to a percept of a square that is bent in depth. This latter result is incompatible with a Bayesian statistics account of what the percept should look like based upon the high probability of experiencing flat squares in the world. These percepts are explained in Grossberg (1997) and simulated in Kelly and Grossberg (2000). In the bottom row (left panel), the two small rectangles are recognized as an amodally completed vertical rectangle behind the horizontal bar. This illustrates amodal completion of recognition without seeing, as do the two stratification figures. This percept, and its variants when the relative contrasts of the rectangles and background are varied, is explained in Grossberg (1997). The remaining figure in the lower right panel illustrates bistable transparency, whereby the percept of an upper left square appears as a transparent film in front of a lower right square alternates with the percept of a lower right square as a transparent film in front of an upper left square. This percept, as well as unimodal transparency and no transparency cases, is explained and simulated in Grossberg and Yazdanbakhsh (2005).

Boundaries need to be completed for several reasons. One is to complete boundaries across the retinal blind spot and veins. Another is to complete the boundaries of partially occluded objects behind their occluders (Figure 4). Both types of completion occur in the visual cortex and project to higher cortical levels, including inferotemporal cortex, where they facilitate recognition of the corresponding objects.

**Figure 5:**
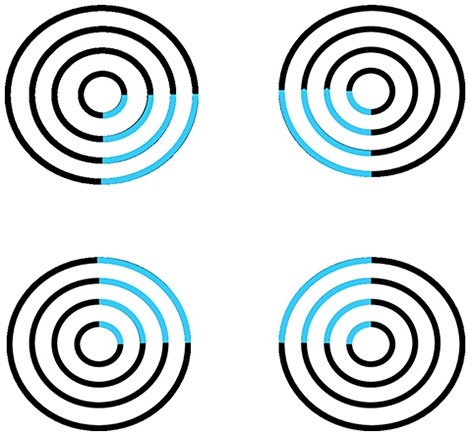
**Neon color spreading**. The blue color in the blue arcs spreads throughout the illusory square. This percept is explained and simulated in Grossberg and Mingolla (1985a).

Surface filling-in proceeds *outwardly* from its inducers in an *unoriented* fashion until it hits a boundary or dissipates due to its spatial spread, as in the percept of neon color spreading in Figure 5. Filling-in occurs in networks that are called Filling-In-DOmains, or FIDOs. Multiple FIDOs exist to enable filling-in of opponent colors (red-green, blue-yellow) and achromatic brightnesses (light-dark) at multiple depths. In each FIDO, filling-in spreads from feature contours that that are computed during a process of “discounting the illuminant” at positions where luminance or color contrasts change quickly enough across space. Such positions often occur along a surface's boundary contours, which act as filling-in barriers. Boundary contours are also sensitive to contrast changes, but use different computations to form.

Feature contours compute brightness and color signals that are significantly invariant under changes in illumination levels. This happens because the contrast changes where they are computed are due primarily to changes in the reflectances of abutting objects, and the illumination level typically changes little, if at all, across such a contrast change. Filling-in spreads illuminant-discounted feature contour signals across the surface until they hit the boundary contours that enclose the surface. The percept of neon color spreading that is seen in response to Figure 5 illustrates how the square illusory contour boundary can prevent the spreading blue color from crossing it. Unlike boundary completion, filling-in is *sensitive* to contrast polarity, consistent with the prediction that “all visible qualia are surface percepts.”

These properties of boundaries and surfaces are complementary: inward vs. outward, oriented vs. unoriented, insensitive vs. sensitive (Figure 1).

Complementary boundary and surface properties are needed for each process to work. For example, filling-in is unoriented to be able to spread over an entire surface. This unoriented flow of brightness or color can, however, only be effectively contained by an oriented boundary. Likewise, a surface seeing process cannot effectively build boundaries around objects in front of textured backgrounds. Both types of process are needed for either process to work well, but they also must interact to overcome each other's complementary deficiencies.

#### Complementary consistency: Surface contours and surface capture

**Figure 6:**
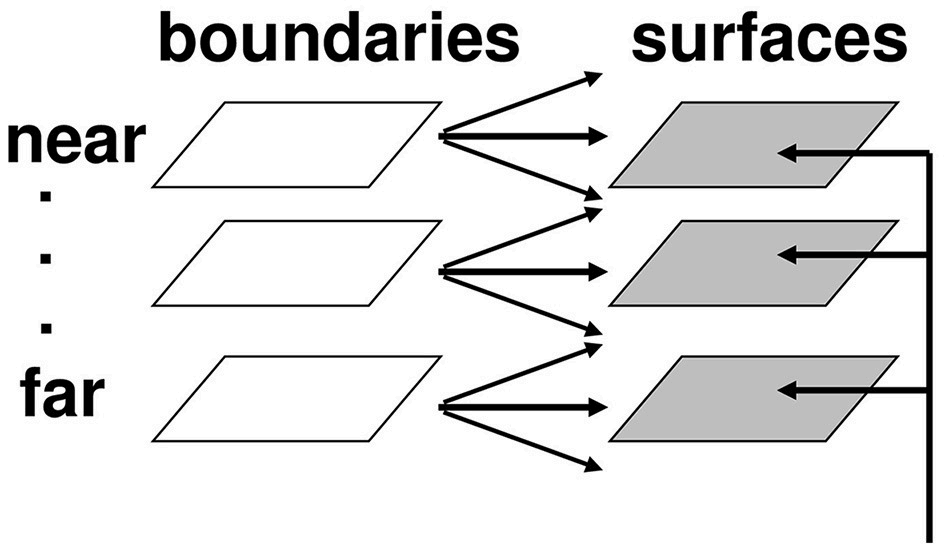
**Multiple depth-selective boundary representations regulate filling-in of surface representations within multiple depth-selective Filling-In DOmains**. Brightness or color feature contour inputs are topographically distributed across multiple depths (vertical arrows) before being captured by boundaries (horizontal and oblique arrows) that are positionally aligned with them. See Grossberg (1994) for a more complete description of this surface capture process.

**Figure 7:**
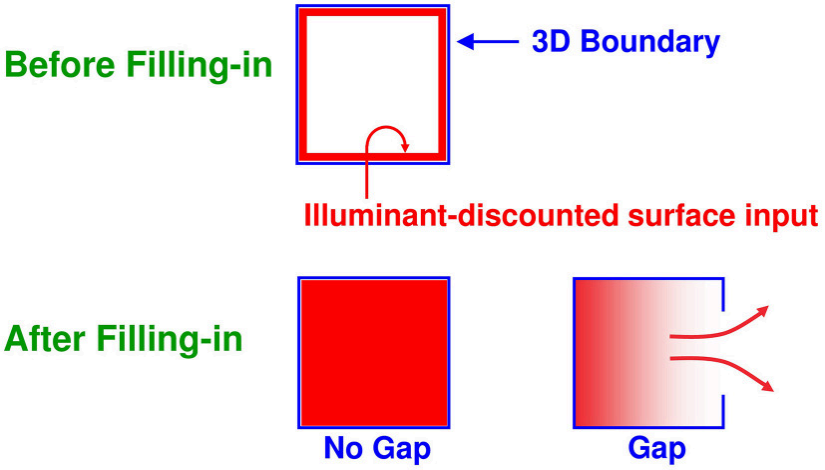
**Filling-in of closed and open boundaries**. The top row illustrates how, at a prescribed depth, a closed boundary contour abuts an illuminant-discounted feature contour. When this happens, the feature contours can fill-in within the closed boundary. The bottom row (left panel) depicts how filling-in of the feature contours is contained by this closed boundary contour, thereby generating large contrasts in filled-in activity at positions along the boundary contour. Contrast-sensitive surface contour output signals can then be generated in response to these large contrasts. The bottom row (right panel) depicts a boundary contour that has a big hole in it at a different depth. Feature contours can spread through such a hole until the filled-in activities on both sides of the boundary equalize, thereby preventing contrast-sensitive surface contour output signals from forming at such boundary positions.

Multiple boundary and surface representations are needed to represent a 3D scene, each sensitive to a different range of depths from an observer (Figure 6). FACADE theory predicts how 3D boundary signals are topographically projected from where they are formed in the V2 interstripes to the surface representations in the V2 thin stripes (Figure 2). These boundaries act as *filling-in generators* that initiate filling-in of surface brightness and color at positions where the boundary contour and feature contour signals are positionally aligned. After filling-in is initiated, boundaries also act as *filling-in barriers* that prevent the filling-in of lightness and color from crossing object boundaries (Grossberg, 1994). If a boundary at a given depth is closed, then it can contain the filling-in of an object's lightness and color within it (Figure 7). If the boundary at a different depth has a sufficiently big gap in it, then surface brightness and color can spread through the gap and surround the boundary on both sides, thereby equalizing the contrasts on both sides of the boundary. Only a closed boundary can contribute to the final visible 3D percept.

**Figure 8:**
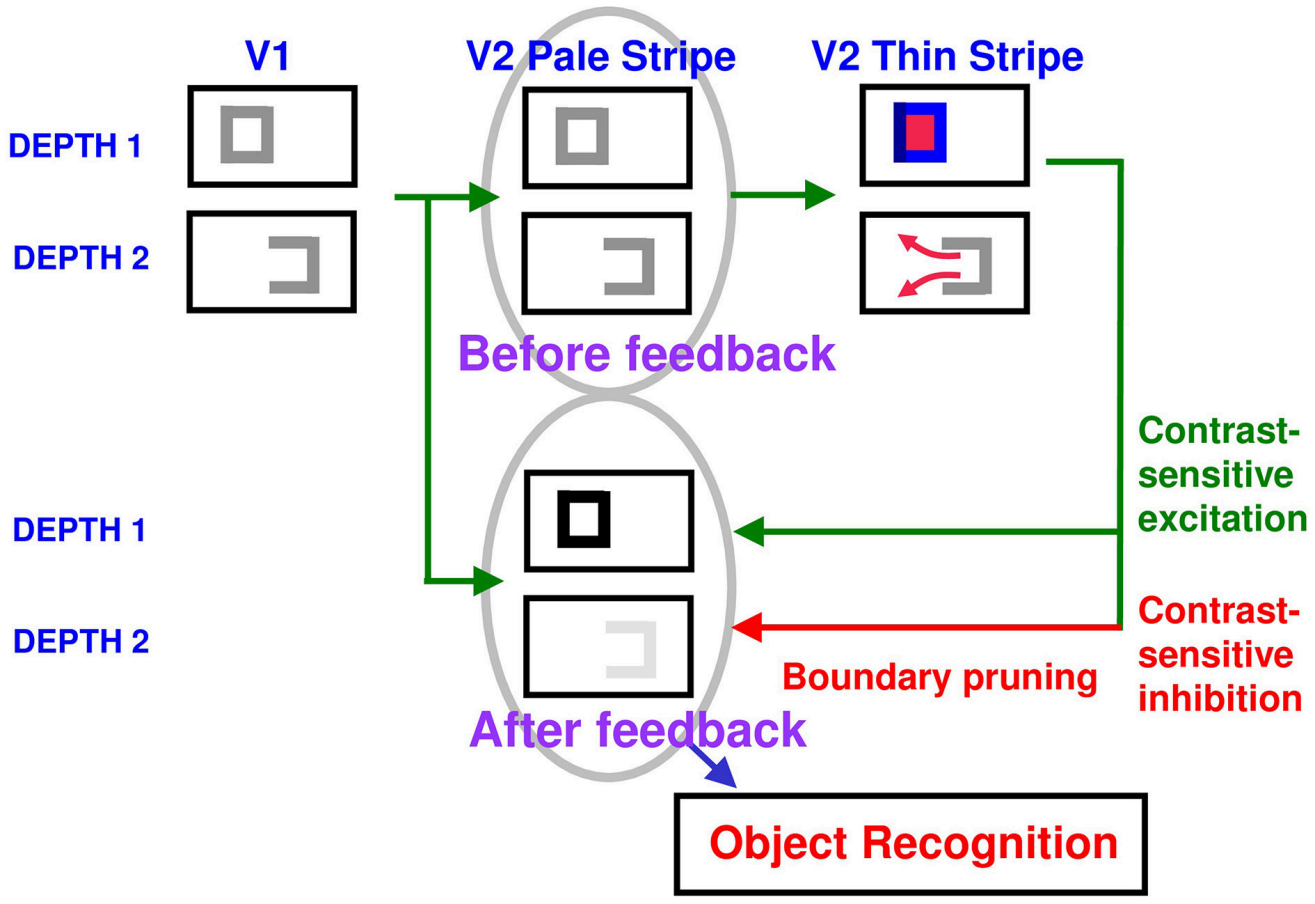
**How closed boundaries regulate seeing and recognition in depth**. A closed boundary can form at the nearer depth Depth 1 by combining a binocular vertical boundary at the left side of the square with three monocular boundaries that are projected along the line of sight to all depths. Surface contour output signals can thus be generated by the FIDO at Depth 1, but not the FIDO at the farther depth Depth 2. The Depth 1 surface contours excite, and thereby strengthen, the boundaries at Depth 1 that controlled filling-in at Depth 1. These surface contours also inhibit the redundant boundaries at Depth 2 at the same positions. As a result, the pruned boundaries across all depths, after the surface contour feedback acts, can project to object recognition networks in inferotemporal cortex to facilitate amodal recognition, without being contaminated by spurious boundaries. See Fang and Grossberg (2009) for simulations of how this process works in response to random dot stereograms.

How do closed boundaries help to form a visible 3D percept? How does this process also help to ensure complementary consistency, and to thereby contribute to figure-ground separation? In addition to the *boundary-to-surface* interactions that act as filling-in generators and barriers, there are also *surface-to-boundary* feedback interactions from filled-in surfaces in V2 thin stripes to the boundaries in V2 interstripes (Figure 8). This feedback is carried out by *surface contour* signals that are generated by a contrast-sensitive on-center off-surround network whose inputs are the filled-in surface activities within each FIDO. The inhibitory connections of this network's off-surround act *across position* and *within depth* to generate contrast-sensitive surface contour output signals from each FIDO. Surface contour signals are hereby generated at positions where sufficiently large changes in brightness or color occur within successfully filled-in surface regions. If the object surface in a FIDO is surrounded by a closed boundary, then there is typically a discontinuity in the contrasts across the object boundary, so surface contours can be generated at these positions. Surface contour signals are not generated at boundary positions near a big boundary gap, since brightnesses and colors can then be equal, hence have zero contrast, on both sides of the boundary due to the spread of filling-in across the gap.

Surface contour output signals generate feedback signals to the boundary representations that induced them. These feedback signals are delivered to the boundary representations by another on-center off-surround network. The inhibitory surface-to-boundary connections of this network act *within position* and *across depth* (Figure 8). The on-center signals strengthen the boundaries that generated the successfully filled-in surfaces. The off-surround signals inhibit spurious boundaries at the same positions but farther depths by a process that is called *boundary pruning*. Surface contour signals hereby achieve complementary consistency by strengthening consistent boundaries and pruning inconsistent boundaries. The inhibited inconsistent boundaries can then contribute to neither seeing nor recognition in the final percept, thereby preventing the perception of spurious percepts of transparency and recognition of irrelevant contour fragments.

Because surface contour signals are generated by the contrast of a filled-in surface, they are sensitive to a particular contrast, and not to the opposite one. Their feedback to boundaries thus makes the boundary cells also sensitive to this contrast, even though the boundaries, in the absence of surface contour feedback signals, pool opposite contrast polarities, starting at V1 complex cells, so that they can complete boundaries of objects in front of textured backgrounds (Grossberg, 1994, 1997).

In the surface contour off-surround networks, inhibitory strength from a surface contour decreases with the distance from the source cell. In the present case, “distance” translates into a depth difference. Thus, the strength of the inhibitory signals *decreases* as the depth difference *increases* between the depth of the surface that generates the surface contour signals and the recipient boundaries.

#### Why brighter Kanizsa squares look closer

Properties of this off-surround network within position and across depth help to explain why, for example, brighter Kanizsa squares look relatively closer in depth than their inducers (Kanizsa, 1955, 1974; Bradley and Dumais, 1984; Purghé and Coren, 1992), as explained more fully in Grossberg (2014). In particular, the brightness of a Kanizsa square increases with the amplitude of the filled-in activity within the square. A larger activity creates larger surface contour signals at each position. These signals are multiplied by the strengths of the inhibitory connections from the signal source to the recipient boundary at the same position but different depths. Due to the decrease in size of the inhibitory connections across depth, these net signals also get smaller as the depth difference increases.

**Figure 9:**
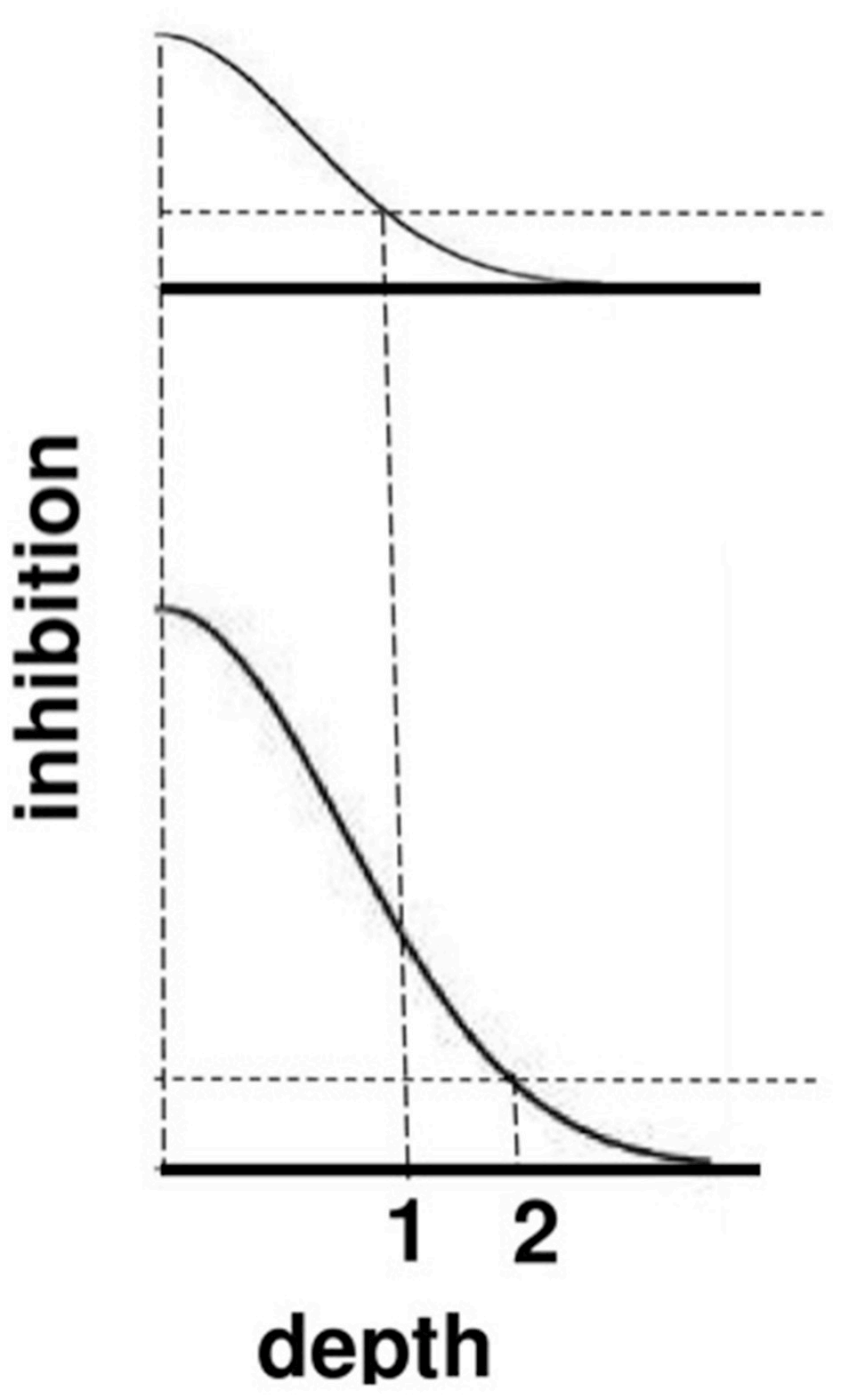
**A cross-section of the inhibitory off-surround across depth that is caused by surface contour outputs**. The top row shows the inhibitory signals in response to a less bright Kanizsa square. The bottom row shows the inhibitory signals in response to a more bright Kanizsa square. The numerals 1 and 2 indicate one of the depths where the inhibitory signals are equal. This illustrates how the brighter Kanizsa square (at depth 1) can inhibit boundaries at more depths between that of the Kanizsa square and its inducers (such as depth 2), thereby making the brighter square stand out more in depth.

The top image in Figure 9 represents the total strength of these inhibitory signals across depth at a lower level of brightness, and the bottom image represents the total inhibitory signals across depth at a higher level of brightness. The two dark horizontal edges depict the x axis that calibrates the depth difference between boundaries for each brightness level, with the upper horizontal edge corresponding to the lower brightness level and the lower horizontal edge corresponding to the higher brightness level. The numbers 1 and 2 indicate that the same level of inhibition is achieved at a larger depth difference in response to a brighter Kanizsa square. A larger number of boundary depths are hereby inhibited by a brighter square than a dimmer one. As a result, the boundary depths that survive well enough to represent the background are more separated in depth from the brighter square than those that survive in response to a dimmer square. In short, brighter Kanizsa squares look closer, relative to their backgrounds, than dimmer ones.

In fact, surface contour signals help to explain many data about visual perception and object recognition. For example, during invariant object category learning, attention shifts, and eye movement scanning of object surfaces, they help to explain how “attention pointers” work (Cavanagh et al., 2010), why the eyes prefer to scan an object's salient features with several successive saccades rather than move randomly around a scene (Theeuwes et al., 2010), and how predictive remapping occurs of cortical receptive fields (Duhamel et al., 1992; Umeno and Goldberg, 1997; Gottlieb et al., 1998; Tolias et al., 2001; Sommer and Wurtz, 2006; Melcher, 2007, 2008, 2009; Saygin and Sereno, 2008; Mathôt and Theeuwes, 2010a,b), all processes that contribute to how the brain learns invariant object categories during free scanning of a scene (Fazl et al., 2009; Cao et al., 2011; Grossberg, 2013).

By eliminating boundaries at depths that do not support visible filled-in surfaces, boundary pruning helps to achieve the process of *surface capture* whereby feature contours can selectively fill-in visible surface qualia at depths where binocular fusion of object boundaries can successfully occur, and can thereby create closed boundaries that can contain the filling-in process. Surface contour and boundary pruning signals hereby work together to generate 3D percepts based on successfully filled-in surface regions.

Importantly, by eliminating spurious boundaries, surface contour signals also initiate figure-ground separation. They do so by enabling occluding and partially occluded surfaces to be separated onto different depth planes, and inhibiting spurious boundaries at the further depths. This process accomplishes two things: (1) It ensures that “border ownership” of the surviving boundaries is inherited by the figural boundaries that generate the surface contour signals. (2) When spurious boundaries at further depths are inhibited, they can no longer interfere with the amodal completion of partially occluded boundaries and surfaces behind their occluders. See Fang and Grossberg (2009), Grossberg (1994), and Kelly and Grossberg (2000) for further details and simulated figure-ground percepts. See Bakin et al. (2000) for experiments in monkeys that describe how amodal contour completion and surface capture may occur in V2.

#### Reasons why boundaries may not be closed: Monocular boundaries and T-junctions

There are several reasons why a boundary may not be closed, so that the brightnesses and colors within them may flow out of the gap in the boundary. Two of them will be summarized here. One reason concerns how monocular boundaries help to form depthful percepts in response to a 3D scene. Another follows from how T-junctions provide a cue to relative depth order for objects in 2D pictures or at far distances in 3D scenes. For distant objects for which binocular disparity is not a useful depth cue, monocular cues, such as T-junctions, may be used to determine relative depth when one object is nearer than another object, and occludes parts of the farther object (Howard and Rogers, 2002).

Some boundaries in a 3D scene may be perceived monocularly during da Vinci stereopsis (Nakayama and Shimojo, 1990; Gillam et al., 1999) where part of the scene may only be seen by one eye due to a nearer surface that occludes that part from viewing by the other eye. Monocular boundaries do not have a definite depth associated with them. How, then, does the brain decide to which depth they should be assigned? A proposed approach to this *Monocular-Binocular Interface Problem* was suggested (Grossberg, 1994, 1997) in order to explain data about 3D figure-ground perception. The same hypothesis was shown by Grossberg and Howe (2003) to help explain many data about 3D surface perception. This hypothesis proposes that the outputs of monocular boundary cells are added to binocular boundary representations at all depth planes in the interstripes of cortical area V2 along their respective lines-of-sight, possibly in layer 4. Yazdanbakhsh and Watanabe (2004) have done psychophysical experiments to test this hypothesis with positive results.

Figure 8 illustrates this hypothesis in response to the image of a square whose right vertical boundary is seen only monocularly due to da Vinci stereopsis. The three-sided boundary at Depth 2 in Figure 8 arises because the vertical boundary is monocular due to da Vinci stereopsis, and the two horizontal boundaries do not generate strong depth information because they do not strongly activate cells that are sensitive to a definite range of binocular disparities. At Depth 1, this three-sided boundary is closed by a fourth vertical boundary that is binocularly viewed in the square, and thereby generates a preferred binocular disparity at Depth 1. The closed boundary at Depth 1 can contain surface filling-in, whereas the open boundary at Depth 2 cannot.

Figure 8 shows how surface contour feedback from the filled-in closed boundary at Depth 1 inhibits redundant boundaries at its positions and further depths, including the open three-sided boundary at Depth 2. Due to this near-to-far inhibition, the depth-ambiguous three-sided boundary is assigned Depth 1 *and* a definite border ownership assignment is also made to the closed boundary at this depth.

**Figure 10:**
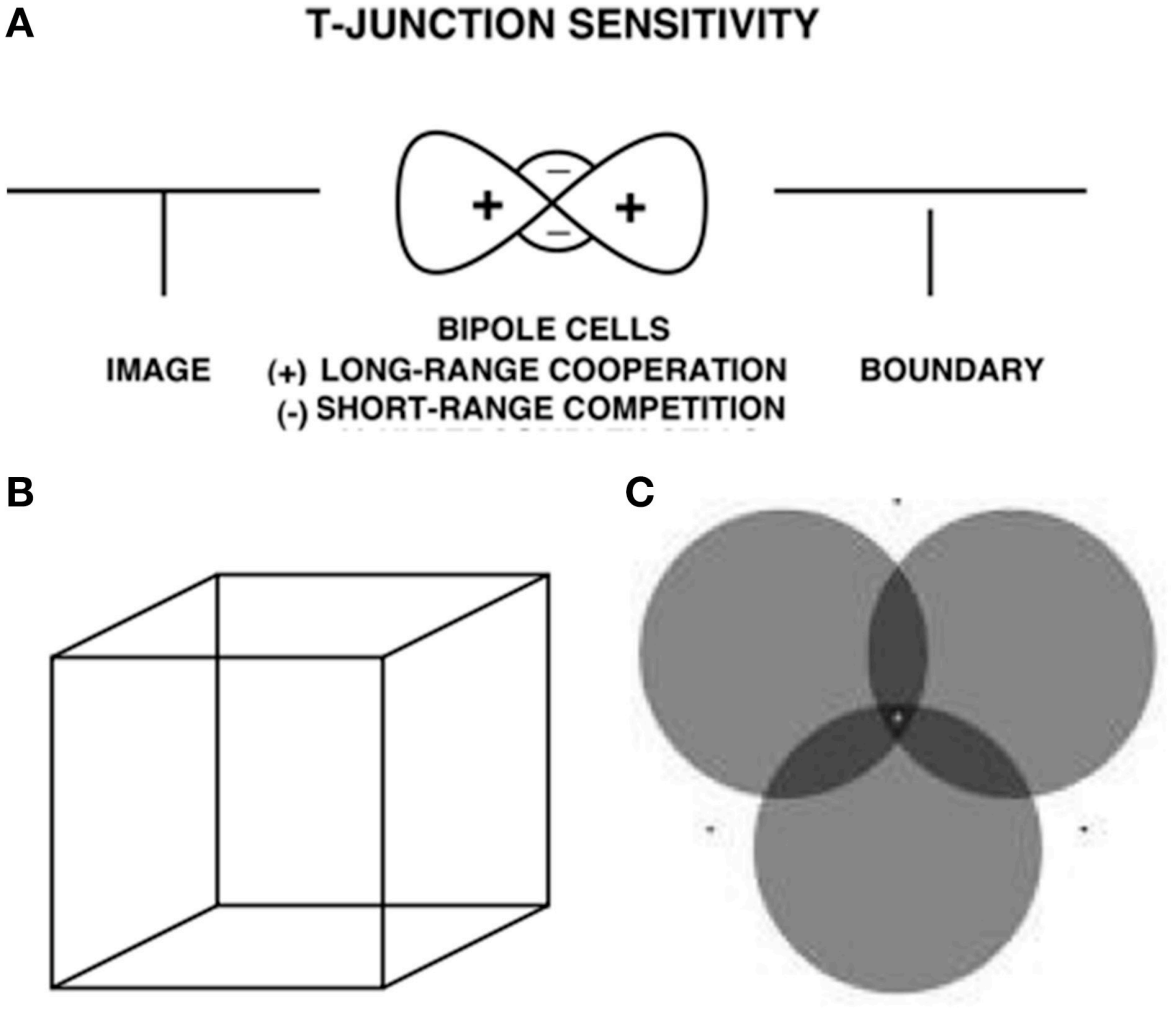
**T-junctions and end gaps in figure-ground perception**. **(A)** T-Junction Sensitivity: (left panel) T-junction in an image. (middle panel) Bipole cells activate long-range recurrent excitatory horizontal connections (cooperation, +) and also short-range inhibitory interneurons (competition, −); (right panel). An end gap in the vertical boundary arises because, for cells near where the horizontal top and vertical stem of the T come together, the top of the T activates bipole cells along the top of the T much more than bipole cells are activated along the T stem. As a result the stem boundary gets inhibited by the short-range inhibitory signals from the horizontal bipole cells, whereas the top boundary does not receive comparable inhibition from the vertical bipole cells (Reprinted with permission from Grossberg, 1997). **(B)** Necker cube. This 2D picture can be perceived as either of two 3D parallelopipeds whose shapes flip bistably through time. **(C)** When attention switches from one circle to another, that circle pops forward as a figure and its brightness changes. See text and Grossberg and Yazdanbakhsh (2005) for an explanation. Reprinted with permission from Tse (2005).

The same surface contour mechanism helps to explain how monocular cues, such as T-junctions, may be used to determine relative depth when one object is nearer than another object, and occludes parts of the further object. This explanation also clarifies how a 3D percept of occluding and occluded objects may be generated in response to a 2D picture that includes such T-junctions. For example, consider the lower left pictorial display in Figure 4. This figure is composed of three abutting rectangles, but it irresistibly generates a 3D percept of a horizontal rectangle that partially occludes a vertical rectangle lying behind it. Here, the horizontal boundaries between the occluding rectangle and its abutting two rectangles are shared. Due to properties of boundary grouping and completion by bipole cells (e.g., Grossberg, 1984, 1994, 1997; von der Heydt et al., 1984; Peterhans and von der Heydt, 1989; Dresp and Grossberg, 1997, 1999; Kelly and Grossberg, 2000; Dresp et al., 2002; Grossberg and Yazdanbakhsh, 2005), the horizontal boundaries are stronger than the abutting vertical boundaries. This happens because horizontally-oriented bipole cells receive inputs from horizontal lines on both sides of each intervening vertical line, whereas the vertically-oriented boundary near their intersection receives inputs from only one vertical line. The horizontally-oriented bipole cells can therefore inhibit the vertically-oriented bipole cells where the horizontal and vertical lines are joined more than conversely, thereby creating small gaps in the vertical boundaries near where they abut the horizontal boundaries (Figure 10A).

These *end gaps* allow brightness and color to flow between the vertical bars and their surrounds during surface filling-in, thereby equalizing the contrasts on both sides of the remaining boundaries near these gaps. Only the boundary of the horizontal rectangle is closed, so only it can contain its surface filling-in, and generate surface contour feedback. All redundant copies of this horizontal rectangular boundary will be inhibited at further depths by near-to-far surface contour inhibition. The boundaries of the two abutting rectangles are spared by this inhibition. As a result, border ownership of the horizontal boundaries by the near occluding rectangle is achieved, and the two rectangles above and below the occluder can complete collinear vertical boundaries and fill-in between them at a further depth, thereby giving rise to a completed vertical rectangle behind the occluding horizontal rectangle. This completed rectangle is used to recognize the two rectangles above and below the occluder as a partially occluded vertical rectangle. Computer simulations of 3D percepts generated by 2D pictures with T-junctions are given in Kelly and Grossberg (2000).

Additional mechanisms are needed to generate the modal, or consciously visible, percepts of the unoccluded parts of both occluding and occluded objects in depth. FACADE theory proposes how boundaries and surfaces may be amodally completed in V2 for purposes of recognition, but that conscious perception of the unoccluded surfaces of opaque objects may be completed in V4. These proposed V2 and V4 representations enable the brain to complete the representations of partially occluded objects behind their occluders without forcing all occluders to appear transparent. See Grossberg (1994, 1997) and Grossberg and Yazdanbakhsh (2005) for further details about how these V2-to-V4 interactions are proposed to work, including computer simulations of opaque and transparent percepts. The present article focuses on properties of V2.

### 2. Transforming absolute disparity in V1 into relative disparity in V2

Cells in visual cortical area V1 are sensitive to *absolute disparity* (Gonzalez and Perez, 1998). Absolute disparity is the horizontal difference in the retinal positions of an image feature that is registered in the left and right foveas after fixation. In contrast, many cells in cortical area V2 are sensitive to *relative disparity* (Thomas et al., 2002). Relative disparity is the difference in absolute disparity of two visible features in the visual field (Cumming and Parker, 1999; Cumming and DeAngelis, 2001), notably of a figure and its background. Absolute disparity varies with distance of an object from an observer. It can change across a visual scene without affecting relative disparity. Indeed, relative disparity, unlike absolute disparity, can be unchanged by the distance of visual stimuli from an observer, or by vergence eye movements that occur as the observer inspects objects at different depths (Miles, 1998; Yang, 2003). Thus, relative disparity is a more invariant measure of an object's depth and its 3-D shape than is absolute disparity.

**Figure 11:**
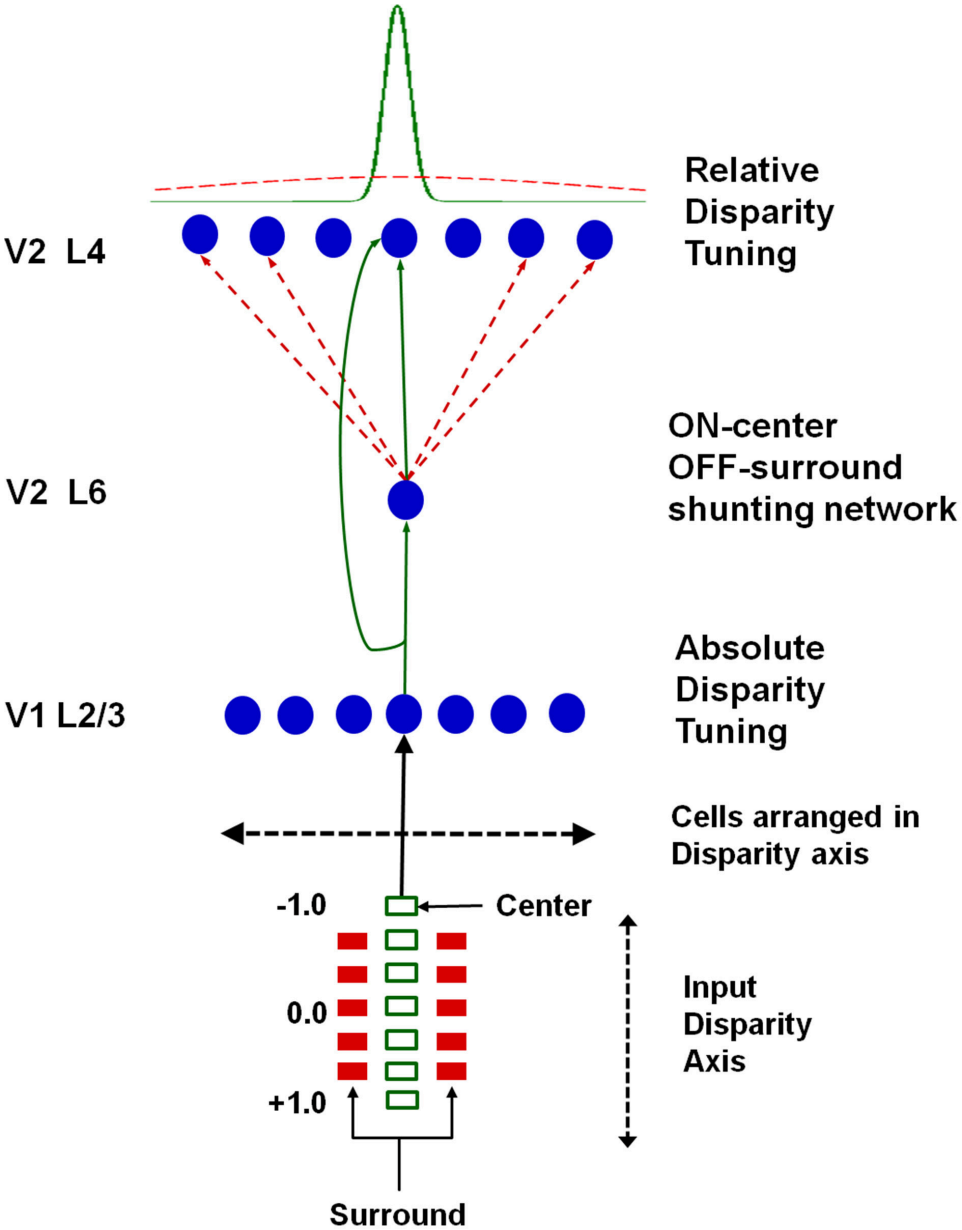
**Model circuit for transforming absolute disparity into relative disparity:** the input consists of dots arranged on a disparity axis along a single position in the plane. The fixation plane is assigned a disparity of 0◦. This input is mapped to complex cells in V1 layer 2/3 that are tuned to absolute disparity and positioned along a disparity axis. The inputs from V1 layer 2/3 to V2 layers 6 and 4 define a shunting on-center off-surround network whose lateral inhibition causes a peak shift in V2 disparity tuning that matches relative disparity data, as illustrated in Figures 12, 13. Reprinted with permission from Grossberg et al. (2011).

**Figure 12:**
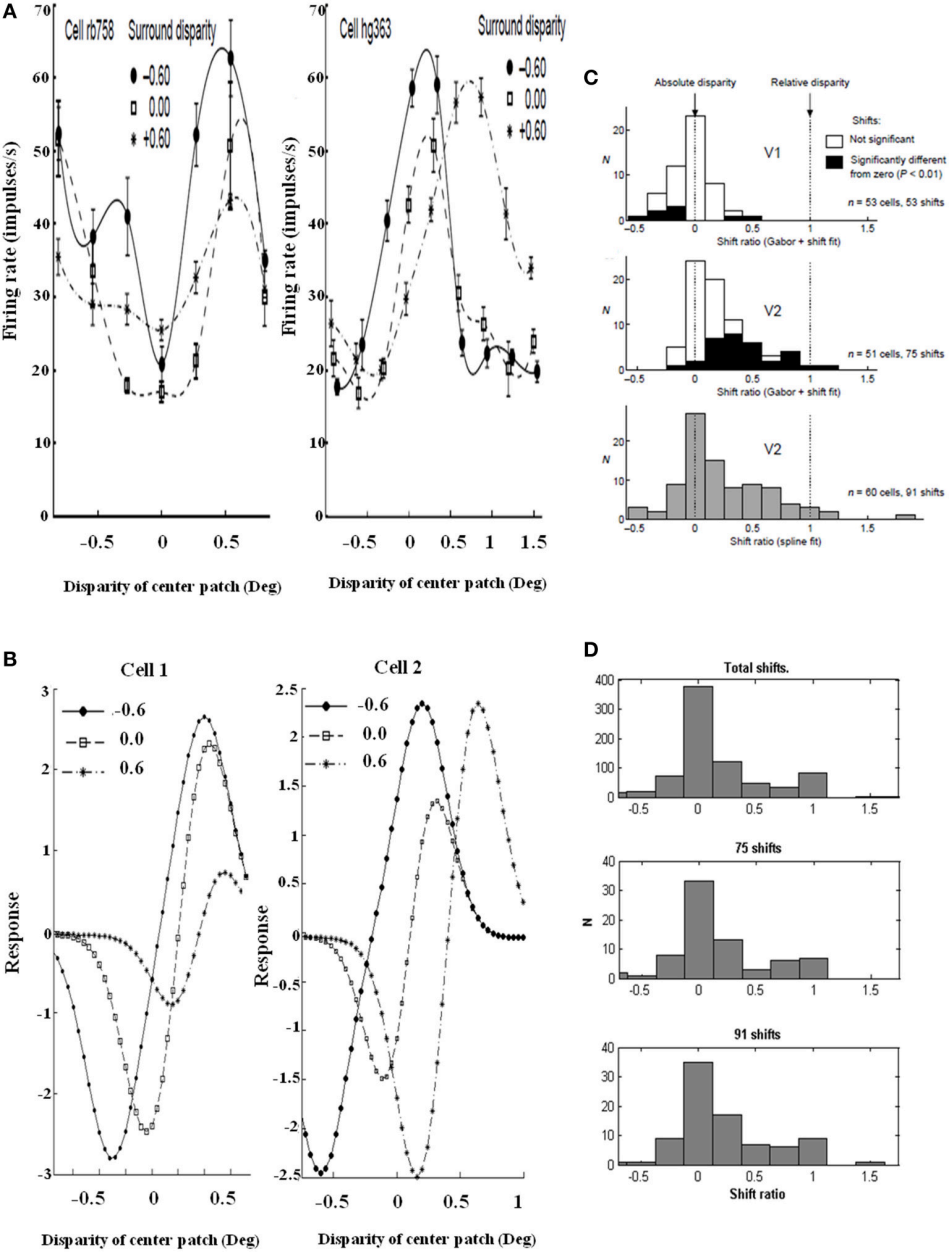
**Relative disparity data and simulations**. (Left panel) Sample cell data from experiments and model: (A) Experimental data of two V2 cell responses for relative disparity (Reprinted with permission from Thomas et al., 2002). **(B)** Two model V2 layer 4 neurons with disparity tuning curves with changes in surround disparity. The model neurons simulate the position of data peaks and their shifts, but not all aspects of the amplitudes in the data. This is due to the simplicity of the model. Despite the simplicity, the model is capable of capturing the key shift properties. (Right panel) Shift ratio statistics. The shift ratio is defined as the shift in peaks of the tuning curve relative to the difference, or shift, of surround disparities. The shift ratio summarizes the statistics of the type of disparity observed: **(C)** Shift ratio summary reprinted with permission from Thomas et al. (2002). **(D)** Shift ratio summary from the model showing best results with *D*^−^ = 0.2 and σ_*inh*_ = 1.0. An exhaustive number of combinations would have required permutations derived from choosing two surrounds without repetition from a set of 200 cells, leading to 19,900 permutations. However, the best available data from Thomas et al. (2002) have a maximum of 91 shifts, so a random selection was compared with their summary statistics. This random selection chose, for each cell, four shift ratios to derive a total of 1600 shifts and 800 shift ratios. These shift ratios were, in turn, randomly sampled without replacement to select 75 and 91 shifts, respectively, to match the number of shifts computed in the experimental data [Reprinted with permission from Grossberg et al. (2011)].

**Figure 13:**
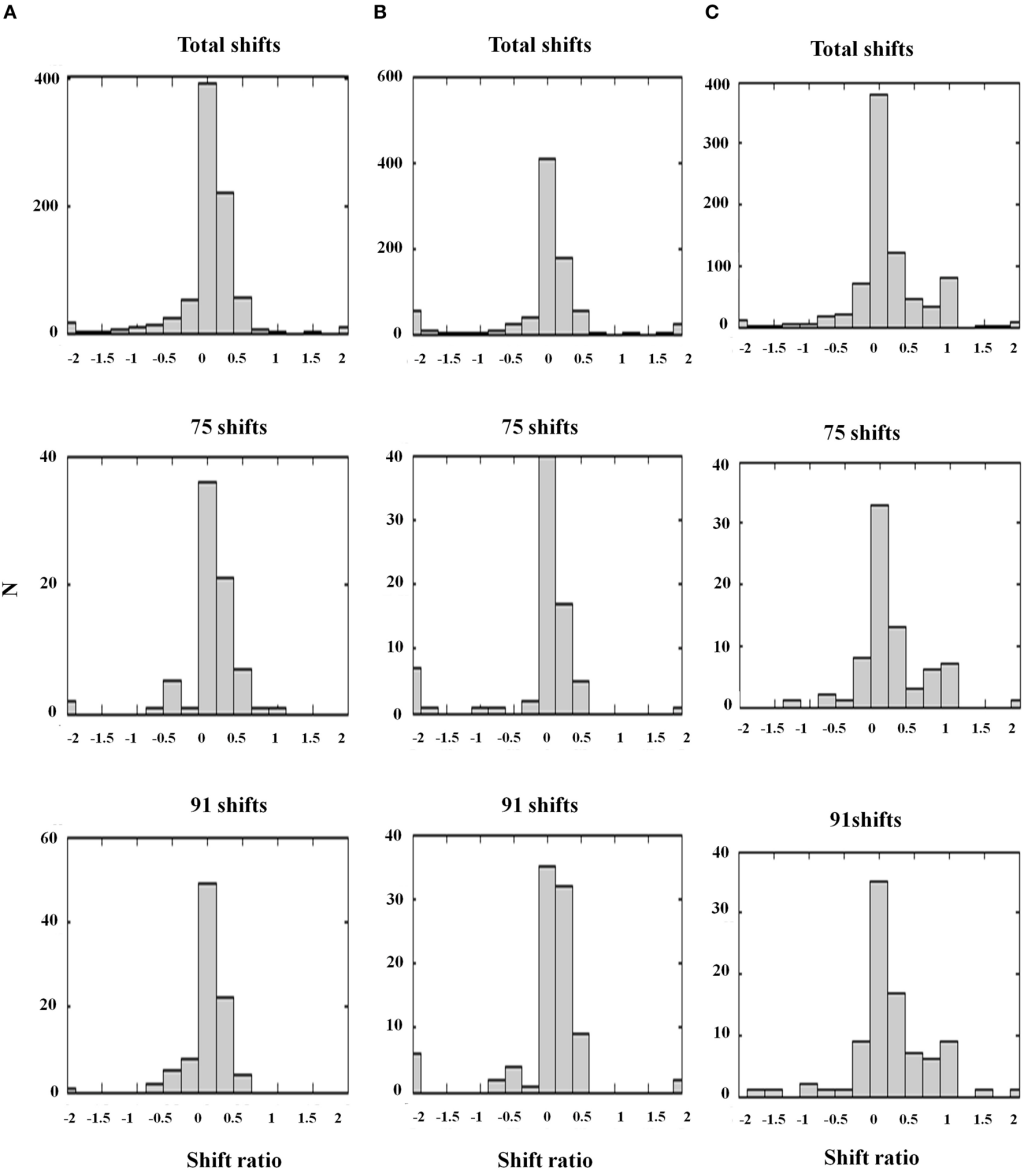
**Shift ratio statistics due to varying *D*^−^ and σ_*inh*_ using the same shift sampling method as in Figure 12**. Shifts toward absolute disparity or relative disparity depend on these parameters. **(A)** *D*^−^ = 0.5; σ_*inh*_ = 1.0. Shift toward absolute disparity. This is the usual profile of V1 disparity cells. **(B)** *D*^−^ = 1.0; σ_*inh*_ = 0.5. Absolute disparity is observed with a larger amplitude and narrower width of the off-surround kernel. **(C)** *D*^−^ = 0.2; σ_*inh*_ = 1.0. A weak amplitude modulation (*D*^−^ = 0.2) and a *wide inhibitory surround* together (σ_*inh*_ = 1.0) generate a gradient from absolute to relative disparity resembling the data. Thus, the nature of the surround inhibition in V1 and V2 accounts for the type of disparity sensitivity, a fact that is important in explaining some figure-ground data of Zhang and von der Heydt (2010); see the text. The parameters used in Figure 12D and **(C)** are the same [Reprinted with permission from Grossberg et al. (2011)].

Grossberg et al. (2011) proposed that the transformation from absolute to relative disparity that occurs between cortical areas V1 and V2 can be achieved by a simple neural model that they used to quantitatively simulate parametric neurophysiological data of Thomas et al. (2002). The model demonstrates that shunting lateral inhibition of layer 4 cells in cortical area V2 can cause a *peak shift* in cell responses (Figure 11). This peak shift is sufficient to transform absolute disparity into relative disparity (Figures 12, 13), thereby creating cells that are sensitive to one or the other side of a figure against its background.

**Figure 14:**
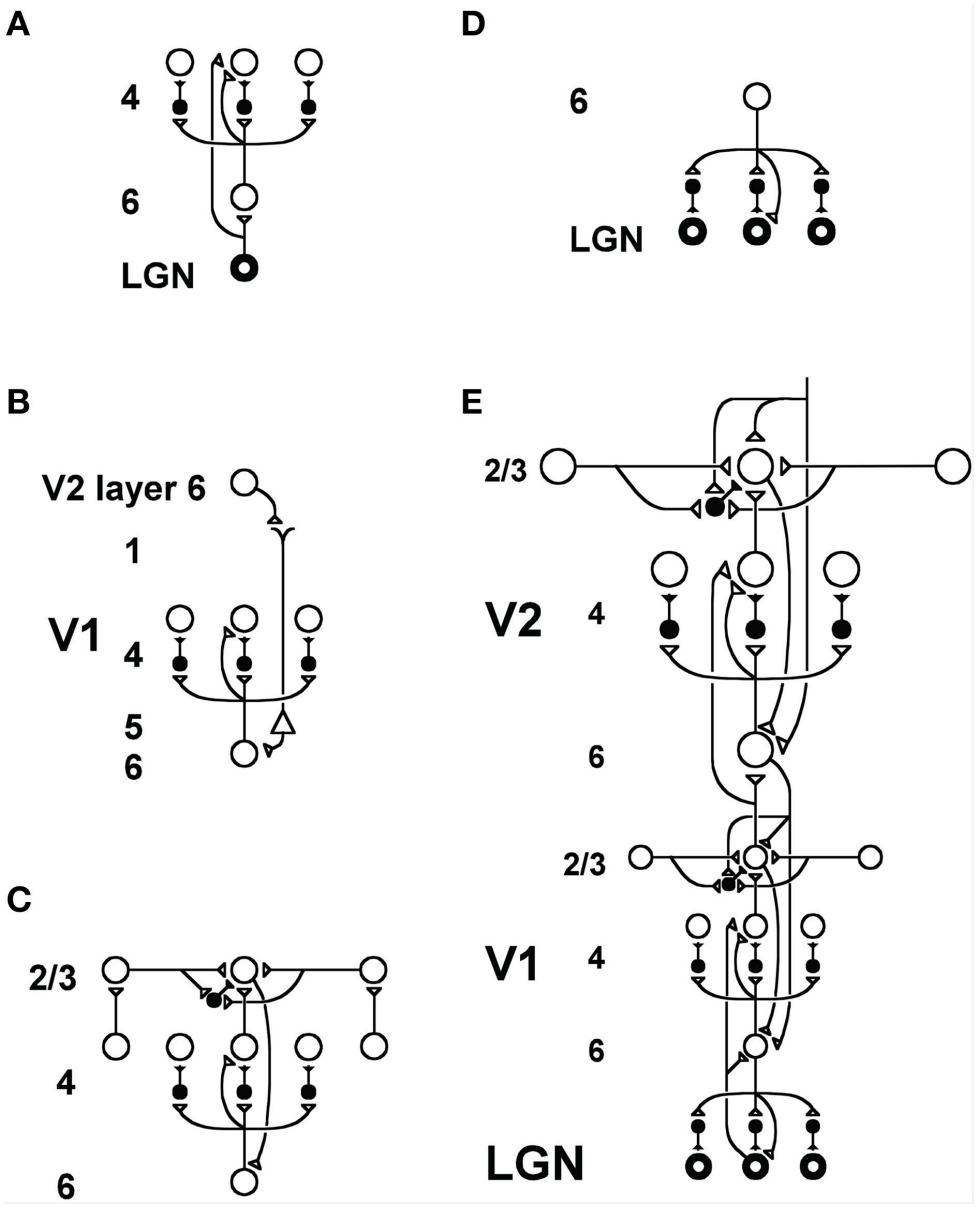
**LAMINART circuitry: (A)** Bottom-up pathways from LGN to V1 layer 4 directly, and via layer 6, together defining a driving shunting on-center off-surround network that contrast-normalizes input patterns. **(B)** Top-down attentional signals from V2 layer 6 reach layer 4 via a “folded feedback” modulatory on-center, off-surround shunting network. **(C)** Horizontal grouping by layer 2/3 bipole cells also engages the layer 6-to-4 decision circuit via a layer 2/3-to-6-to-4 feedback loop to choose winning groupings in response to ambiguous input patterns that may support multiple groupings initially in time. **(D)** Top-down attentional feedback from layer 6 in V1 to LGN also obeys a modulatory on-center, off-surround shunting network. **(E)** Bottom-up, horizontal, and top-down circuits are combined in V1 and V2, with V2 horizontal connections spanning a larger spatial scale. Bottom-up V1-to-V2 layer 6-to-4 off-surround circuits help to convert absolute disparity cells in V1 into relative disparity cells in V2. The circuits in V1 layers 4, 3B, and 2/3 that compute absolute disparity are not shown. See Grossberg and Howe (2003) or Cao and Grossberg (2005) for an exposition of how they work. Reprinted with permission from Raizada and Grossberg (2001).

It is important to realize that this same inhibitory circuit has also been used to explain perceptual and neurobiological data from cortical areas V1 and V2 about contrast gain control and divisive normalization (Figures 14A,E), attentional focusing (Figures 14B,E), and selection of perceptual groupings (Figures 14C,E), as explained in Grossberg (1999) and simulated in Grossberg and Raizada (2000). Thus, the lateral inhibition mechanism within cortical area V2 in Figure 11 that is capable of transforming absolute into relative disparity is a known anatomical feature of V2 and was modeled in the LAMINART model of 2D perceptual grouping and attention (Figure 14E). Grossberg et al. (2011) showed that this known inhibitory mechanism can also explain data about V2 relative disparity cells in the full 3D LAMINART model. The model hereby shows how relative disparity interacts with other visual functions and thereby suggests new experimental manipulations for testing these functional relationships.

## Methods

### 3. Unifying two streams of theory: The role of modulatory learned feedback connections

The transformation of absolute disparity into relative disparity in V2, as described in Section 2, endows these V2 cells with a preference for a definite depth order of figure and ground, and also to a preference for a definite side-of-figure. This is, however, a relatively local transformation, dependent as it is on a local lateral inhibition circuit that affects individual V2 cells. The figure-ground mechanisms that were summarized in Section 1, in contrast, involve more global transformations that involve entire figures and their backgrounds, and the Gestalt laws that may be explained by boundary grouping and surface filling-in processes, and their interactions, within and between these figures.

In response to bottom-up signals from a figure in a 3D scene, relative disparity cells in V2 can code for the depth order of figure and ground and preference for side-of-figure. If different figures occur at different relative depths from an observer, and are close enough to the observer for binocular disparities to separate them in depth, then the *unoccluded* boundaries of these figures will automatically “belong” to them (“border ownership”) entirely due to binocular disparity processing. However, even in this case, if some of the figures are partially occluded by nearer figures, then binocular disparities are not sufficient to amodally complete the boundary and surface representations of partially occluded surfaces behind their occluders. As noted in Figures 8, 9, feedback between the V2 boundary and surface representations, notably surface-to-boundary surface contour feedback signals, are needed to inhibit redundant boundaries of occluding figures at further depths, thereby freeing the unoccluded boundaries of occluded figures to be completed behind their occluders, and also to fill-in the partially occluded surfaces there. These surface contour feedback signals complete the border ownership assignment at the occluded positions of further surfaces.

An important property of these surface contour feedback signals is that they can operate quickly. Indeed, both the boundary and surface representations are directly activated by bottom-up inputs. In cases where the object boundaries in the scenes are complete, bottom-up activation of boundaries is sufficient to activate all object boundary positions in the V2 interstripes, before horizontal bipole interactions have a chance to confirm and bind these positions together into coherent boundary groupings that obey Gestalt laws. These horizonal interactions can act much more quickly when bottom-up activations are already in place than they can when completing a boundary over empty space.

Bottom-up boundary activations can also quickly activate boundary-to-surface filling-in generators and barriers while the horizontal bipole groupings are starting their work. These boundary-to-surface signals trigger rapid filling-in of closed boundaries within the V2 thin stripe surface representations. As soon as this filling-in process creates a sufficient threshold level of contrast difference at surface contours, surface contour feedback signals can rapidly be generated back to their inducing boundaries, simultaneously at all boundary positions, thereby completing the border ownership assignment at the occluded boundaries.

Figure-ground separation and border ownership dynamics in response to 2D pictures with T-junctions use similar mechanisms. Again, bottom-up inputs activate the V2 boundary and surface representations. Because a 2D picture is viewed at a particular depth during normal 3D vision, disparity-sensitive cells may be activated during these initial boundary activations, but there is not yet any evidence about figure and ground during this first activation sweep. As soon as end gaps are formed at T-junctions, however, and filling-in can distinguish closed vs. open boundaries, surface contour feedback signals can separate the closed figures in depth, thereby activating relative disparity boundary cells with their side-of-figure preferences and globally realizing border ownership assignments.

**Figure 15:**
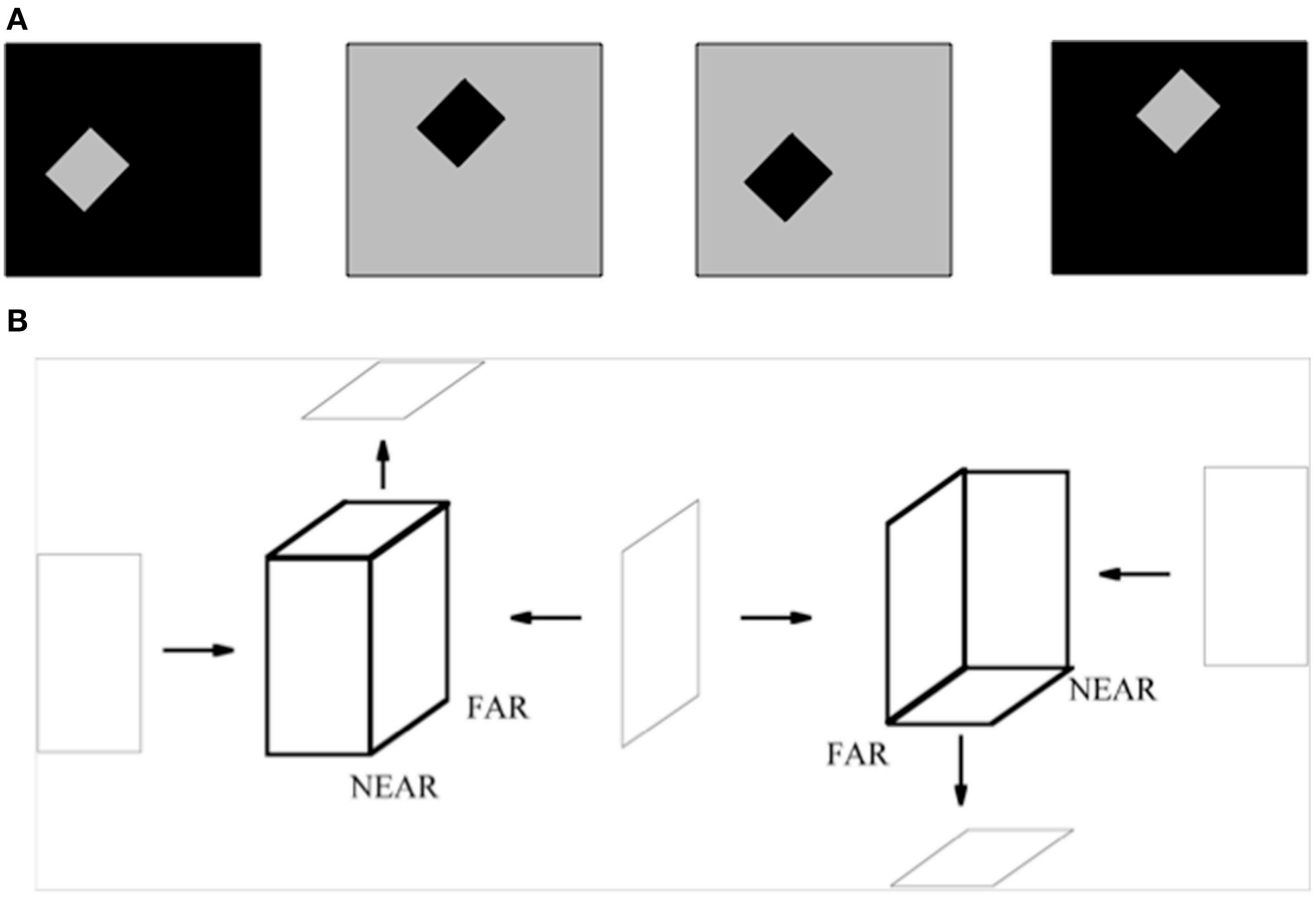
**(A)** The standard test for determining the effect of border ownership on edge responses. In the left two images, identical contrast edges are presented in the recorded receptive field. In the left-most figure, the light-dark edge is at the right side of a light square. In the figure to its right, it is at the left side of a dark square. The relation is analogous between the right two images, with reversed contrasts. Adapted from Zhou et al. (2000). **(B)** 2D pictures can appear flat or slanted in depth. The two figures in bold lines are made of same set of surfaces, but due to the different arrangement of their surfaces, they give rise to different slanted 3D percepts, even though the figural sides from which they are composed can individually be perceived as flat. The left bold figure has a positive tilt (near-to-far), while the right bold figure has a negative tilt (far-to-near). The tilts of near-to-far vs. far-to-near straight lines are determined by the combinations of angle cells between which they lie. The corresponding combinations of surrounding angle cells and straight bipole cells are associated with each other during normal 3D vision. Reprinted with permission from Grossberg and Swaminathan (2004).

A small square or rectangle with a uniform contrast in a larger uniform background may also be perceived as a figure on a ground (Figure 15A). In this case, as well, surface contour feedback signals strengthen their generative boundaries in the small figure. Although disparity-sensitive cells may be activated when such a 2D picture is viewed in 3D space, there is no explicit depth cue in the figure of Figure 15A to separate such a figure from its background, or depth-inducing trigger such as a T-junction. How, then, is such an image perceived as a figure against a background?

Computer simulations in Grossberg et al. (2002) clarify how surface contour feedback signals can selectively activate relative disparity cells due to prior learning with 3D objects during cortical development under normal 3D viewing conditions. Indeed, the inter-stream connections between the boundary and surface streams need to be tuned by learning during perceptual experiences in order to properly align them. For example, in V1, the complex cells that help to form V1 interblob boundaries are binocular, hence subject to allelotropia, or displacement, on the cortical map. This displacement occurs because an external cue causes inputs to the left and right eyes that are positionally displaced due to binocular disparity. When a complex cell binocularly fuses the positionally disparate signals on the cortical map that these monocular inputs cause, allelotropia can occur.

In contrast, the color and brightness cells in the V1 blobs that receive inputs corresponding to these monocular left and right eye inputs are monocular, and thus are not displaced due to binocular disparity.

Learning is needed to enable interblob and blob cells that represent the same position in space to input to each another. Grossberg et al. (2002) simulated how such positionally-specific inter-stream learning can be accomplished, and used these learned connections to quantitatively simulate data about the McCullough effect, which is an orientation-contingent complementary color aftereffect that is typically induced by several minutes of adaptation to gratings of black and color stripes, and that can last for hours, days, or even weeks. Indeed, all the explanations of boundary-surface interactions within the FACADE and 3D LAMINART models depend upon such positionally-specific learned connections in order to work.

In the computer simulations of Grossberg et al. (2002), the surface-to-boundary feedback is *modulatory*. It *multiples*, and thus gain-controls, the bottom-up signals and thereby enhances them, but it cannot activate the target cells on its own. Modulatory cross-stream signals also occur in models of how form information can modulate the processing of motion information; e.g., Berzhanskaya et al. (2007). Such modulatory connections are, more generally, predicted by Adaptive Resonance Theory to be a ubiquitous mechanism for learning self-stabilizing associations (see Grossberg, 2013 for a review).

But how does this modulatory surface contour feedback know, in response to the rectangular figure in Figure 15A, what side of the rectangle these relative disparity cells should code. This property is clarified by computer simulations of Grossberg and Swaminathan (2004) which showed how learning occurs during cortical development between pairs of V2 angle cells, that occur at the corners of a shape like the rectangle, and the collinear bipole cells between them. Such learning typically occurs during experiences with 3D objects under normal 3D viewing conditions. After such learning occurs, it can disambiguate the flat, near-to-far, or far-to-near depth interpretations of many different kinds of ambiguous geometric figures in 2D pictures (e.g., Figure 15B). In Grossberg and Swaminathan (2004), such learned connections were used to simulate how a 2D picture of a Necker cube (Figure 10B) can generate a pair of 3D surface representations that oscillate bistably through time to generate the two distinct conscious 3D surface percepts that are characteristic of this striking visual illusion.

These simulations of context-sensitive perceptual learning call attention to the importance of scenic features like corners in disambiguating the depths of their intervening smooth contours. In particular, the fact that the two angle cells that bound each straight edge of the rectangle have a consistent interpretation as part of the figure forces the corresponding boundary-to-boundary associations to form among relative disparity cells along that edge. These associations, in themselves, provide a strong cue to select consistent relative disparity cells along the entire figural boundary, thereby creating a 3D representation of the figural boundary as being in front of its background. The boundary-to-surface-to-boundary feedback loop can then complete the surface representations of the figure and background to be consistent with this boundary representation.

When these two kinds of learning simultaneously operate in response to 3D scenes, they generate several important properties. The first property is that surface-to-boundary learned connections can initially start to be formed with *all* the relative disparity cells that are simultaneously active with them. Due to their high correlation during perception of 3D objects, strong learned associations can form between the surface contour signals and the relative disparity boundary cell activities that code for the same position and orientation. The associations between surface contour signals and relative disparity signals that code for other positions tend to wash out due to associative interference during the viewing of many different objects. These strong learned surface contour associations include the layer 6-to-4 inhibitory interneurons that help to activate relative disparity cells. The simulations of Grossberg et al. (2011) fit the data of Thomas et al. (2002) under the assumption that inhibitory off-surround is broad and is defined using the gain-control properties of shunting competition. Thus, a surface contour activation to one side of the figure could inhibit the surface contour activation of the other side of the figure through this lateral inhibitory network, and several simultaneous surface contour effects of this kind could partially normalize each other due to the shunting competition.

One further observation is that the 3D LAMINART circuits for top-down attention also engage the layer 6-to-4 pathway via top-down signals that reach layer 6 from layer 1 (Figures 14B,E; Grossberg, 1999). Data and simulations supporting such a “folded feedback” attentional circuit are described in Grossberg and Raizada (2000) and Raizada and Grossberg (2001). By this shared pathway, top-down attentional signals can support percepts of figure vs. background via the relative disparity cells.

In summary, a combination of learning processes that go on during normal 3D vision can disambiguate a figure from its background and activate the correct relative disparity cells along the rectangular boundary, including their positional, orientational, border ownership, contrast-sensitive, side-of-figure, Gestalt grouping, and attentional properties. These learning processes include contextually-sensitive (angle cell)-(colinear bipole cell) associations within the boundary stream, supplemented by learned surface-to-boundary surface contour associations.

## Results

### 4. Neurophysiological data and model explanations about figure-ground perception, border ownership, and attention

In their classic article about the coding of border ownership in monkey visual cortex, Zhou et al. (2000) identified cells in cortical area V2 that responded selectively to the combination of a particular image contrast at a surface border in a 2D picture, as well as to the border ownership property of being at the left side or right side of the surface (Figure 15A). For example, the same light-dark edge (dark-light edge) of a region could be on the left side (right side) of a dark square or the right side (left side) of a light square. Many V2 border ownership cells responded selectively to the combination of contrast and border position. Other cells were sensitive to one or the other property, but not both. The border ownership properties emerged less than 25 ms after response onset, and these various properties were nearly independent of surface size. How this combination of contrast-sensitivity and border ownership properties could arise in figures of this type, as well as its fast action, was explained in Section 3.

Zhou et al. (2000) also found similar properties using random dot stereogram displays. Here edges were always perceived as belonging to the nearer surface, a property that was explained and simulated using the 3D LAMINART model in simulations of random dot stereogram percepts by Fang and Grossberg (2009).

Zhang and von der Heydt (2010) extended this study to the case of fragmented figures, notably fragmented rectangles, that were constructed from Cornsweet edges. It should be noted that such Cornsweet edges, despite sometimes not being closed, were constructed from contrasts that gradually changed across space and that were visible after boundary completion and surface filling-in, hence could create surface contours. The surround fragments produced facilitation on the preferred border ownership side as well as a rather uniform distribution of surround suppression on the non-preferred side. The authors proposed that these surround effects could be understood as the combined effect of gain normalization and a border ownership mechanism that produces symmetrical enhancement and suppression on the two sides. In addition, fragments far from the recorded cell's classical receptive field influenced responses without extra delays. The authors concluded from this that “the antagonist surround influences are produced by reentrant signals from a higher-level area” (p. 1). They also compared border ownership effects obtained with solid figures and Cornsweet figures having the same geometry, and concluded that Cornsweet figures were almost as effective as solid figures in generating border ownership signals. This result showed that border ownership modulation “depends more on the contours than on regions of different color or luminance” (p. 11), consistent with the hypothesized role of surface contours in generating border ownership signals.

These results, including the gain normalization and rapid action of the fragments, are consistent with the way in which surface contour signals from V2 thin stripes (Figure 8) are proposed to activate the broad off-surround in V2 interstripes that helps to define relative disparity cells (Figure 13). The 3D LAMINART model proposes that the “reentrant signals from a higher-level area” are actually mediated by feedback signals between V2 thin stripes and interstripes, a possibility that was not considered in the von der Heydt et al. articles.

von der Heydt et al. (2000) considered how the processing of stereoscopic edges is related to border ownership preferences within individual V2 neurons. They noted that (p. 1955) “While cells in V1 generally responded according to the disparity of the surface at the receptive field, we found cells in area V2 that responded selectively to the figure edges. These cells signaled the location and orientation of contrast borders as well as stereoscopic edges, and were often selective for the direction of the step in depth.” These cells, moreover, tend to be orientation-selective and exhibit foreground/background, or figure-ground, selectivity, generally preferring near disparities, corresponding to the fact that occluding contours belong to a foreground object and thus tend to have a near disparity. The authors also noted that (p. 1965) “This means that features of 3D objects would evoke relatively constant neural signals even in the presence of variations in object distance, or variations in convergence of the eyes.” These properties are consistent with properties of V2 relative disparity cells, and indeed von der Heydt et al. (2000) discussed the experimental results of Cumming and Parker (1999) on relative disparity, but also noted differences in experimental protocols between the two classes of experiments that limit the extent to which comparisons can be made. These results are consistent with FACADE and 3D LAMINART model results showing how V2 cells become sensitive to relative disparity (Section 2), and how they interact across the V2 boundary and surface streams to carry out figure-ground separation (Section 1), as further specified in Section 3.

Qiu and von der Heydt (2005) further noted that (p. 155) “area V2 combines two strategies of computation, one that exploits binocular stereoscopic information for the definition of local depth order, and another that exploits the global configuration of contours (Gestalt factors). These are combined in single neurons so that the “near” side of the preferred 3D edge generally coincides with the preferred side-of-figure in 2D displays. Thus, area V2 represents the borders of 2D figures as edges of surfaces, as if the figures were objects in 3D space. Even in 3D displays, Gestalt factors influence the responses and can enhance or null the stereoscopic depth information.” These results are compatible with model properties about how the cortical mechanisms of 3D vision enable the brain to interpret 2D pictures as representations of a 3D scene, and about how processes of perceptual grouping, which have elsewhere been shown to give rise to properties of Gestalt laws (e.g., Grossberg et al., 1997), play a key role in figure-ground perception of both 3D scenes and 2D pictures. In particular, Qiu and von der Heydt (2005, p. 163) also note that “The influence of global configuration is still mysterious. Our results show that the range of this influence extends far beyond the limits of the classical receptive fields, which might be taken as indicating a process of central origin. However, several observations argue against this possibility…One is the early differentiation of the responses for the two sides of the figure…[another] is that the side-of-figure preference of each single neuron is fixed in relation to its receptive field. Another neuron with the same location and orientation of receptive field may have the opposite preference. This means that the identification of the figure area is probably not due to an influence of top-down attention.”

Sections 1–3 propose how these properties naturally arise due to inter-stream interactions between the V2 interstripes and thin stripes. In particular, surface contour feedback signals within V2 act upon an entire figure at the same time to achieve complementary consistency and figure-ground separation. The feedback loop that exists between the V2 boundary and surface representations, which includes the surface contour feedback signals from surface to boundary representations, is also consistent with data of O'Herron and von der Heydt (2009, p. 801) showing that “figure-ground signals in the visual cortex can persist for a second or more after the removal of the figure-ground display. When new figure-ground information is presented, the signals adjust rapidly, but when a figure display is changed to an ambiguous edge display, the signals decay slowly.” The positive feedback signals between the V2 interstripe boundaries and stripe surfaces constitute such a short-term memory. Indeed, Francis and Grossberg (1996) and Francis et al. (1994) have simulated how visual persistence, and its reset, can be controlled in a figure-selective manner using the FACADE model. These modeling results quantitatively simulate the amount of persistence that has been reported in response to a variety of psychophysical displays. It would be of interest to combine such classical psychophysical manipulations of persistence with figure-ground displays while recording from both V2 interstripe and thin stripe neurons to better test the neural mechanisms whereby persistence is regulated in the visual cortex.

Qiu et al. (2007) studied how top-down attention interacts with figure-ground properties in V2, and found that attentional modulation was stronger when the attended figure was located on the neuron's preferred side of border ownership. These data are consistent with the fact, noted in Section 3, that relative disparity and attentional computations both seem to engage the same layer 6-to-4 shunting competitive circuit, but even more so with computer simulations using the 3D LAMINART model to demonstrate interactions between attention and figure-ground mechanisms. Some simulations have shown how attention can influence which of the bistable 3D representations of the Necker cube (Figure 10B) is perceived (Grossberg and Swaminathan, 2004). Other simulations have shown how attention can reverse which of two transparent percepts is seen in front during percepts of bistable transparency [Figure 4 (lower right panel); Grossberg and Yazdanbakhsh, 2005]. These latter results also explain how perceived depth covaries with perceived brightness in displays such as those reported by Tse (2005); see Figure 10C. These latter simulations would be particularly useful as guides to new experiments where 3D boundary groupings are reorganized by a shift in the attentional focus, leading to a corresponding reorganization in the boundary groupings that control surface filling-in.

Both of the above types of simulations consider the effects of *boundary attention*; namely, the kind of attention that can flow along a boundary grouping, thereby strengthening the boundary's activities, as was first reported by Roelfsema et al. (1998). Simulations by the LAMINART model of the interaction between attention and boundary groupings were first provided in Grossberg and Raizada (2000) for the case of 2D percepts, and were extended to the case of 3D percepts in Grossberg and Swaminathan (2004) and Grossberg and Yazdanbakhsh (2005). The model proposes how top-down attentional signals can enter cortex in layer 1 (Figure 14B) and then prime layer 4 cells via a layer 6-to-4 route. Perceptual groupings, supported by bottom-up inputs from layer 4-to-2/3, can propagate along layer 2/3 horizontal bipole cell connections as they also activate a feedback loop between layers 2/3-to-6-to-4-to-2/3 (Figure 14C), including the same 6-to-4 circuit that has been attentionally primed. When the combined attentional priming and boundary grouping signals summate, this enhanced activity can continue to propagate along the perceptual grouping.

The ability of attention to propagate along a boundary grouping raises interesting, and heretofore experimentally untested, predictions about how attention can influence which 3D percept of the Necker cube will be perceived (Figure 10B). In particular, suppose that attention focuses on a Necker cube line that is not located at an X-junction where two lines intersect. As attention spreads along the grouping, it eventually reaches an X-junction. By strengthening the boundary of one branch of the X-junction, it breaks the boundary of the other branch near the intersection of the lines, causing end gaps in the line. End gaps were first used to explain how color can flow out of line ends during percepts of neon color spreading (Grossberg and Mingolla, 1985a). It later was shown by the FACADE model (Grossberg, 1994; Grossberg and Yazdanbakhsh, 2005) how they could also trigger figure-ground perception and explain percepts of 3D neon color spreading (Nakayama et al., 1990), among others. The interesting new experimental question is: Given that the depth ordering that is generated by attention at an X-junction of a Necker cube is totally ambiguous away from the X-junction, and can trigger a definite depth ordering only through the relative strengths of the lines at the X-junction (see also Dresp et al., 2002), how are border ownership cells with a definite figure-ground preference activated in such a situation? Sections 1 and 3 propose an answer that can be experimentally tested.

It should also be noted that, by creating end gaps, attention enables brightness or color to flow between the regions that were previously separated by the broken line, thereby explaining the Tse (2005) data about covariation of attention, figure-ground preference, and brightness (Figure 10C) in the manner simulated in Grossberg and Yazdanbakhsh (2005).

*Surface attention* also plays an important role in figure-ground perception. The ARTSCAN family of models builds upon 3D LAMINART as a front end to explain how invariant object categories are learned as the eyes freely scan a 3D scene, and how these categories can be used to search for a valued goal object in the scene, as in the Where's Waldo problem (Fazl et al., 2009; Grossberg, 2009; Cao et al., 2011; Foley et al., 2012; Chang et al., 2014; Grossberg et al., 2014). Surface attention, in the form of an *attentional shroud* that fits itself to the shape of the attended surface, helps to regulate what object views are linked to the same invariant object category by associative learning between posterior inferotemporal cortex (ITp) and anterior inferotemporal (ITa) cortex as the eyes freely scan the scene. Such an attentional shroud is maintained by a s*urface-shroud resonance* that is predicted to be generated by feedback between prestriate visual cortical area V4 and the posterior parietal cortex (PPC), and to then propagate both top-down to earlier cortical areas and bottom-up to higher cortical areas while spatial attention focuses on the object surface. Such top-down spatial attention enhances the perceived contrast of the attended surface, as has been reported both psychophysically (e.g., Carrasco et al., 2000) and neurophysiologically (e.g., Reynolds and Desimone, 2003). This enhanced surface contrast generates stronger surface contours, which feed back to their generative boundary groupings, thereby strengthening them as well, and altering the course of figure-ground perception accordingly.

It is also of interest to note the prediction that conscious percepts of visual qualia are triggered by surface-shroud resonances (Grossberg, 2013), a hypothesis that helps to explain properties of parietal neglect (Driver and Mattingley, 1998; Mesulam, 1999) and perceptual crowding (Bouma, 1970, 1973; Toet and Levi, 1992; Green and Bavelier, 2007; Levi, 2008; Foley et al., 2012), among other phenomena. Figure-ground separation plays an important role in determining what shrouds can form by separating object surfaces so that spatial attention can, in fact, focus on separated objects in depth. These results on how spatial attention influences figure-ground perception, and its relationships to invariant object category learning, eye movement search, and visual consciousness, also provide fertile new ground for experimental tests.

## Discussion

### 5. Other figure-ground models

Zhang and von der Heydt (2010) showed that most other available models of figure-ground and border ownership properties failed to explain one or more key properties of their data. They also suggested that the model of Craft et al. (2007) from their own group might do a better job of this. However, this model also has problems explaining the data, some of which are summarized in Zhang and von der Heydt (2010). One general problem that is not mentioned by Zhang and von der Heydt (2010) concerns the core model assumption that “contour signals are integrated by neurons at a higher level of the cortex, and BOS selectivity is created by feedback to V2.” However, such feedback to V2 would be just the kind of pathway that subserves top-down attention, and seems to be contradicted by the work of Qiu and von der Heydt (2005) showing that “the identification of the figure area is probably not due to an influence of top-down attention.” A more serious problem concerns whether the proposed mechanism can work in response to scenes with multiple objects in them. Figure 3 in Craft et al. (2007) schematizes the bottom-up and top-down interactions of their model, including signals from a single higher-order cell to multiple positions along the border of a figure. It is unclear how the top-down signals know what positions in the figure they should be selectively exciting or inhibiting. This problem arises even when considering a single figure, but seems to become totally unmanageable when one considers the thousands of figures that would need to get positionally precise top-down feedback from such a circuit. This problem does not arise in the current proposal because a surface contour feedback signal only needs to learn how to activate a boundary at its own position, and this kind of point-to-point boundary-surface learning has already been demonstrated by model simulations, as in simulations of the McCollough effect (Grossberg et al., 2002).

### 6. Conclusion

The 3D LAMINART model has, in previous articles, explained, simulated, and predicted many perceptual and neurobiological data about how the visual cortex carries out 3D vision and figure-ground perception, and how these cortical mechanisms enable 2D pictures to generate 3D percepts of occluding and occluded objects. These explanations clarify how identified cells in laminar circuits of several visual cortical areas interact to generate emergent properties that map onto these diverse perceptual data. The current article extends these explanations to explain all the main properties of cortical area V2 cells that have been reported in a remarkable series of neurophysiological experiments by von der Heydt and his colleagues. These properties include border ownership, contrast preference, binocular stereoscopic information, selectivity for side-of-figure, Gestalt rules, and strength of attentional modulation, as well as the time course during which such properties arise. These explanations go beyond the V2 neurophysiological data to predict how these properties contribute to the generation of consciously seen 3D surfaces. Of equal importance is the fact that these properties of figure-ground separation arise naturally from basic neural principles about how inter-stream interactions between computationally complementary boundary and surface mechanisms are converted into a consistent conscious percept of a 3D surface, and thereby automatically give rise to properties of figure-ground separation. This conversion from complementarity to consistency critically uses the surface contour feedback signals from surface representations in V2 thin stripes to boundary representations in V2 interstripes that play such an important role in our explanations of figure-ground data. The circuit design that converts absolute disparity in V1 to relative disparity in V2 is equally important, and also has other functional roles, such as contrast normalization of bottom-up signals, choice of horizontal perceptual groupings, and modulation by top-down attention. Whatever refinements and revisions the future may bring to these explanations, it therefore seems that some of their fundamental design principles are here to stay.

### Conflict of interest statement

The author declares that the research was conducted in the absence of any commercial or financial relationships that could be construed as a potential conflict of interest.
